# Could Panitumumab with very low dose Capecitabine be an option as a maintenance regimen

**DOI:** 10.18632/oncotarget.28687

**Published:** 2025-02-12

**Authors:** Doaa A. Gamal, Aiat Morsy, Mervat Omar

**Affiliations:** ^1^Department of Clinical Oncology, Assiut University Hospital, Assiut 71515, Egypt

**Keywords:** Panitumumab, maintenance, colorectal cancer, Capecitabine

## Abstract

Background: Anti–epidermal growth factor receptor therapy showed an overall median survival improvement in *wild type Ras* metastatic colorectal cancer. Maintenance with anti *EGFR* in metastatic colorectal cancer wild type *Ras* was studied in many trials with promising results and many of these trials gave combined chemo with the target therapy and this combination had shown benefit in the form of synergistic effect and in delaying the resistance to the anti *EGFR*.

Method: In our study patients received 6 cycles of 5-FU based chemotherapy with Panitumumab and patients who had partial response, complete response or stationary disease received metronomic Capecitabine with Panitumumab every 2 weeks for one year. The primary end point was progression free survival (PFS) and the secondary end points were safety, toxicity and overall survival (OS).

Results: The median PFS for all patients was 18 ± 1.4 months and the median OS was 45 months. Patients with synchronous metastasis and those who received Oxaliplatin based regimen with Panitumumab were found to have longer PFS compared to those with metachronous metastasis or those who received other chemotherapy regimen with accepted toxicity profile to the maintenance therapy.

Conclusion: Using Panitumumab with metronomic Capecitabine is considered an accepted maintenance regimen in wild type *Ras* metastatic colorectal cancer regardless of the primary site.

## INTRODUCTION

Colorectal cancer is the 7th commonest cancer in Egypt, representing 3.47% of male cancers and 3% of female cancers [1]. The standard first-line treatment for metastatic colorectal cancer is fluorouracil-based chemotherapy plus anti–epidermal growth factor receptor/vascular endothelial growth factor (anti-EGFR/VEGF) therapy showing overall median survival of 29 to 30 months [2–3], with switching to a low-intensity or low-toxicity maintenance therapy in responding patients trying to reach a balance between the clinical efficacy and adverse effects (AEs) [4].

Considerations around maintenance therapy are of particular importance when drugs associated with cumulative neurotoxicity like Oxaliplatin form part of adopted regimens. Accumulating toxicity can cause treatment discontinuation and negatively impact the quality of life. In light of such issues, stop-go and/or maintenance strategies have been proposed [5]. The MACRO, MACRO-2, and NORDIC-VII trials also concluded that single-agent Bevacizumab or Cetuximab maintenance therapy was more tolerable than continued induction therapy. In addition, the CAIRO3 trial found that Bevacizumab combined with Capecitabine for maintenance therapy have better results compared with an observation group, suggesting that combination could achieve maximal clinical efficacy [6–8]. Panitumumab is a human anti-epidermal growth factor receptor (EGFR) monoclonal antibody indicated in the treatment of patients with wild type Ras (WT) metastatic colorectal cancer (mCRC), Panitumumab works by binding to the extracellular domain of the EGFR preventing its activation. This results in halting the cascade of intracellular signals dependent on this receptor like RAS/RAF pathway resulting in increased apoptosis, decreased proliferation and angiogenesis [9].

Capecitabine is a cell cycle specific for S phase. It is a prodrug converted enzymatically to 5-FU which inhibits DNA synthesis and slows the growth of tumor tissue. So synergistic effect is expected when combining the two drugs and adding chemotherapy could overcome resistance to panitumumab or prolong the time of resistance [10]. Phase III PRIME study (NCT00364013) and Phase II PEAK study (NCT00819780), both assessed the use of Panitumumab as part of Oxaliplatin-containing first-line therapy [11–14]. To date, the role of Panitumumab with metronomic Capecitabine in maintenance therapy has not yet been properly investigated according to our knowledge. This study primary end point is progression free survival for mCRC cases that received maintenance Panitumumab plus metronomic Capecitabine.

## RESULTS

This study involved 25 patients with metastatic colorectal cancer who achieved CR and PR on Panitumumab based combination and then received maintenance Panitumumab/Capecitabine. The median age of those patients was 50 years. Female patients have the largest percentage of participation, and 88% of the patients had PS of 0-1, Table 1.

**Table 1:** demographic data of 25 patients

Demographic data	Descriptive
Age, median	50 years
Min-max	35–82
Mean ± SD	54.4 ± 13.1
Gender ratio	1:2.1
Male	8 (32%)
Female	17 (68%)
ECOG-PS	
0	15 (60%)
1	7 (28%)
2	3 (12%)

Data expressed as number, mean ± SD, median, and percentage.

Mucinous carcinoma was reported in 20% of the patients, and adenocarcinoma represented 80%. Grade 2 was encountered in >50% of the patients, T3–T4 lesions were reported in 92% and N1-2 in 72% of patients. Most patients were left-sided and had single organ site for metastasis, Table 2.

**Table 2:** Clinicopathologic criteria of 25 patients with advanced colorectal cancer

Criterion	Descriptive
Pathological subtype	
Adenocarcinoma	20 (80%)
Mucinous carcinoma	5 (20%)
Grade	
1	2 (8%)
2	14 (56%)
3	9 (36%)
T-stage	
T2	2 (8%)
T3	12 (48%)
T4	11 (44%)
N-stage	
0	7 (28%)
1	11 (44%)
2	7 (28%)
Primary site	
Rt side	8 (32%)
Lt side	17 (68%)
Site of metastasis	
Single organ metastasis	14 (56%)
Two organ metastasis	5 (20%)
More than two organ metastases	6 (24%)
Timing of metastasis	
Synchronous	13 (52%)
Metachronous	12 (48%)

Data expressed as number and percentage. Distribution of treatment is according to the time of metastasis.

### Distribution of treatment according to the time of metastasis

**Figure 1:**
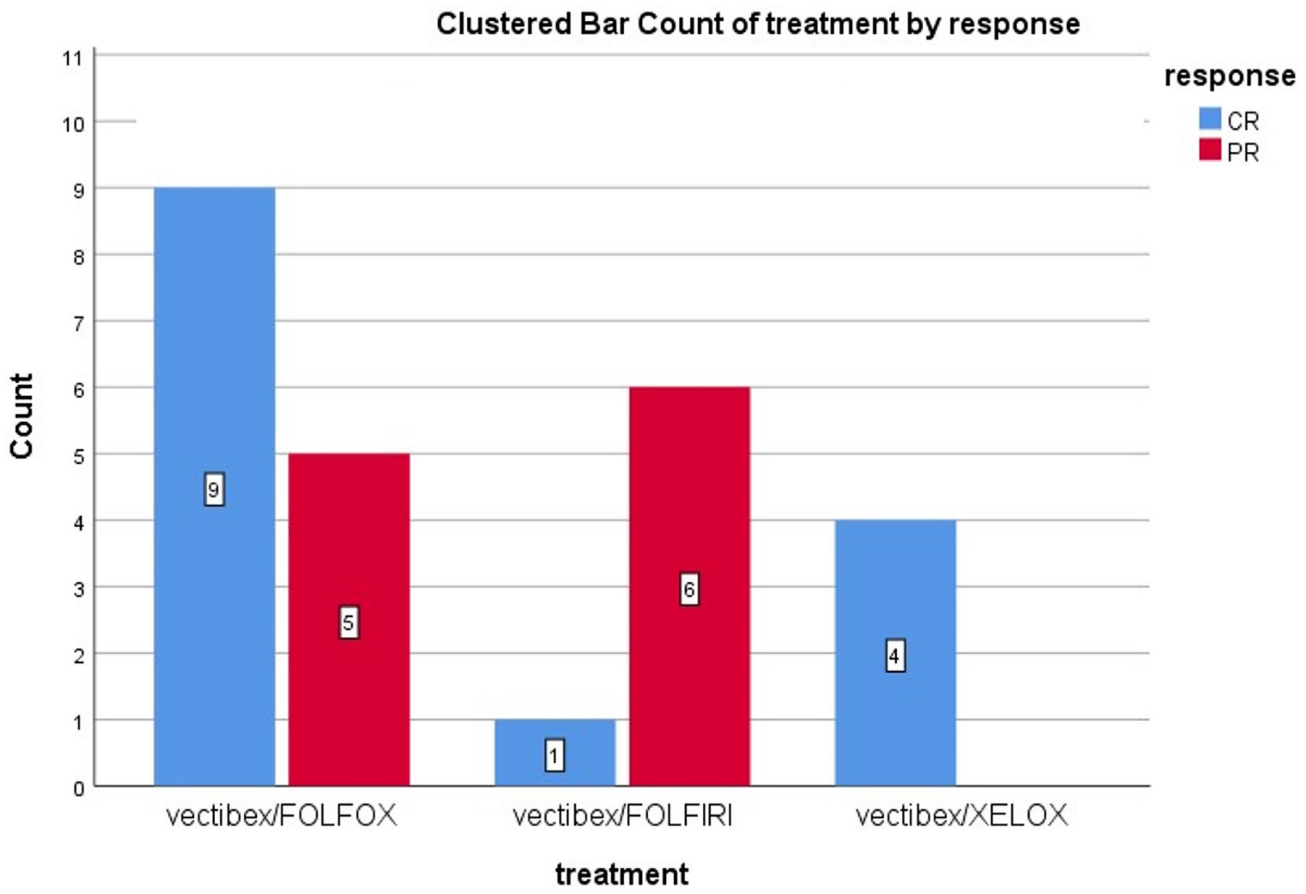
Differences in response type according to treatment, likelihood ratio = 10.3, *p* = 0.006.

Most patients with synchronous metastases received Panitumumab/FOLFOX followed by Panitumumab/Capox then Panitumumab/FOLFIRI, while no one of those with metachronous metastasis received Panitumumab/Capox, and they were equally divided between the other two lines with significant impact (*p* = 0.008) as shown in Figure 1.

### Treatment and response of patients

First-line treatment for metastatic patients was reported to be Panitumumab/FOLFOX, Panitumumab/Capox, and Panitumumab/FOLFIRI in 56%, 16%, and 28% of the patients respectively, which resulted in a CR rate of 56% (14 patients) and a PR rate of 44% (11 patients). Interestingly, most patients receiving Panitumumab/FOLFOX, Panitumumab/Capox achieved CR compared to Panitumumab/FOLFIRI with a significant difference (*p* = 0.006), Figure 1.

### Survival functions

**Figure 2:**
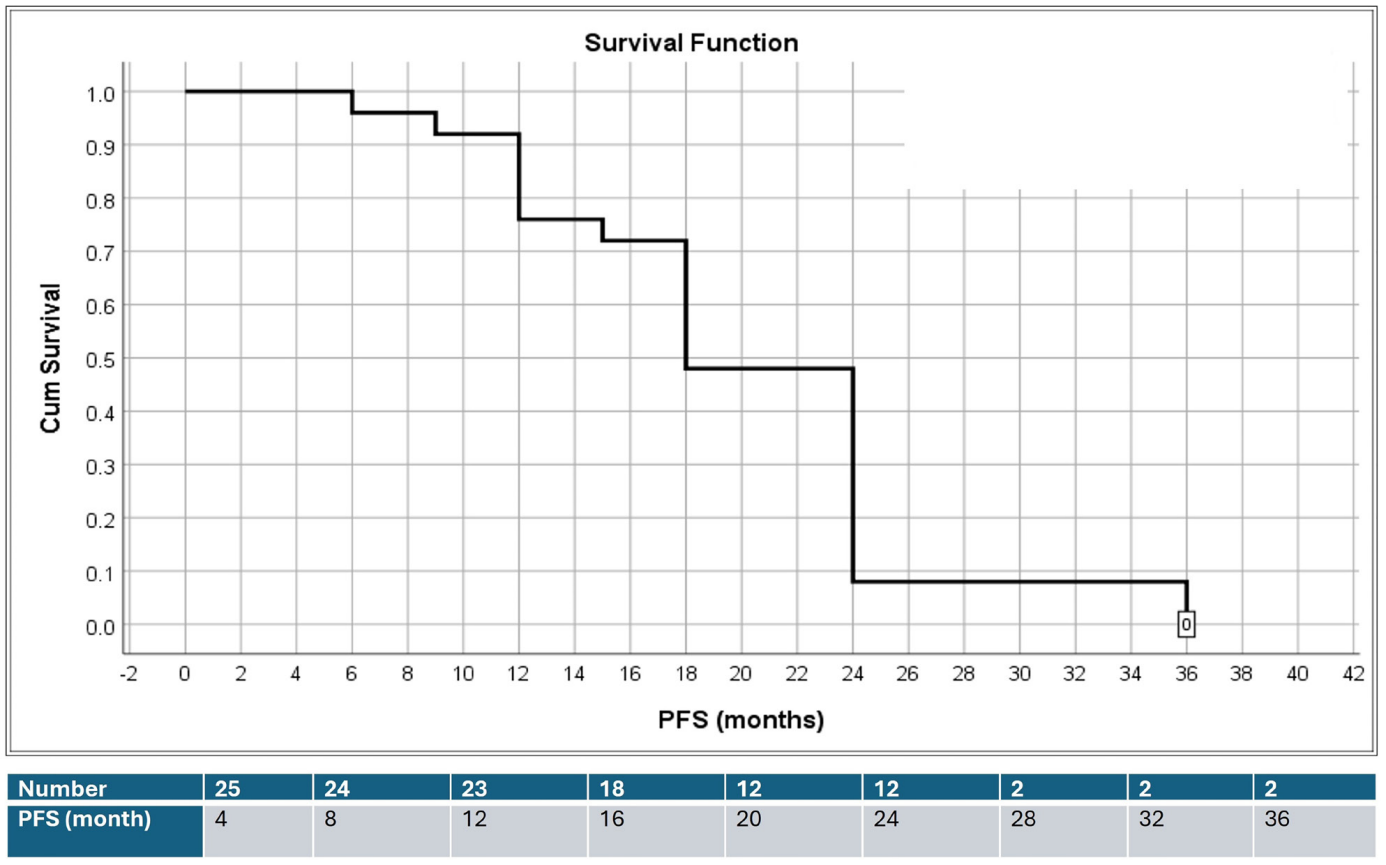
PFS in 25 patients with advanced colorectal cancer.

The median PFS for all patients, whether they were dead, progressed, or censored, was 18 ± 1.4 months (95% CI = 15.3–20.8), Figure 2.

**Figure 3:**
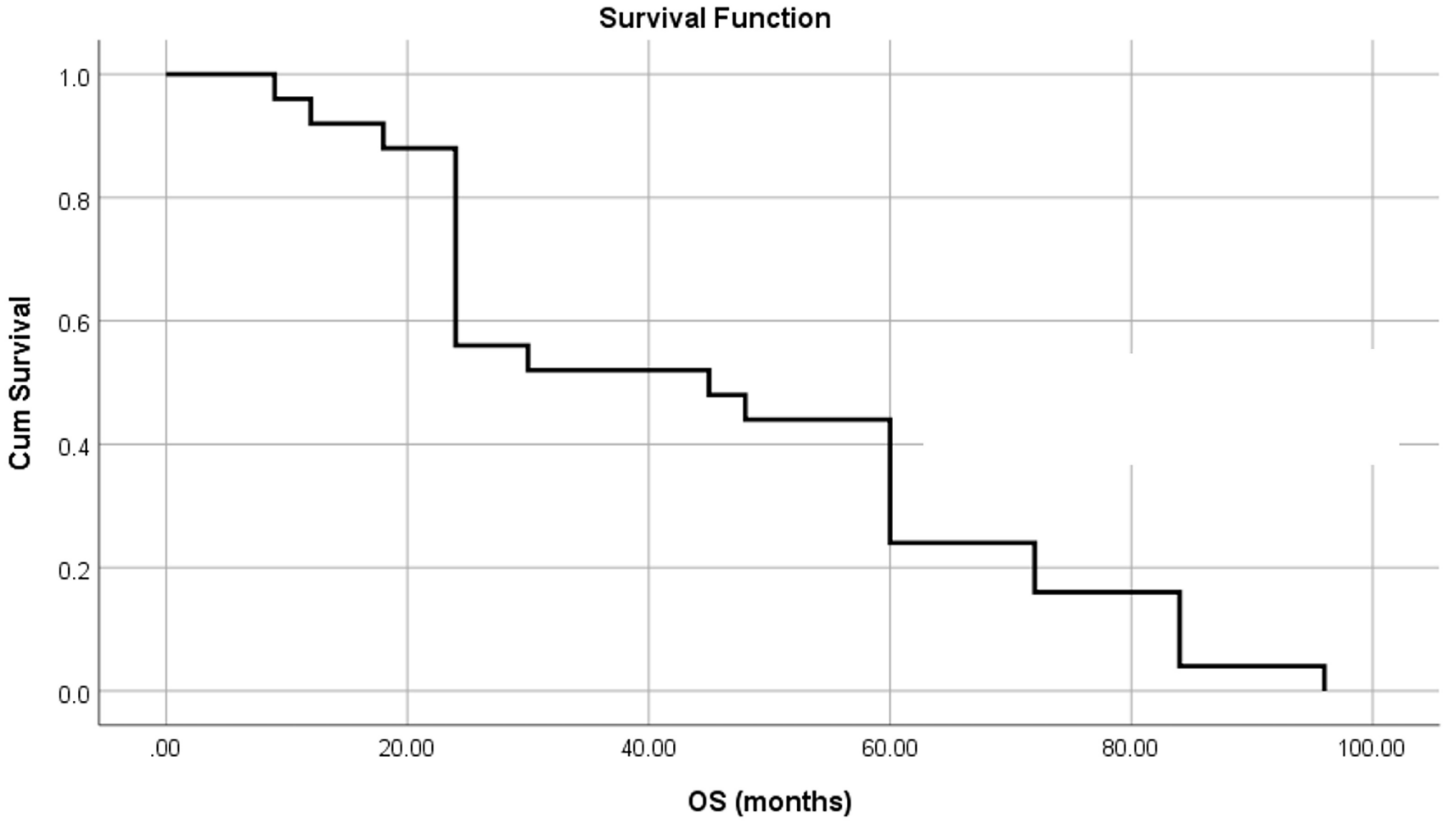
OS for all patients.

The mean OS was 45.8 ± 5.3 months (95% CI = 35.5–56.2) and the median OS was 45 months, Figure 3.

**Figure 4:**
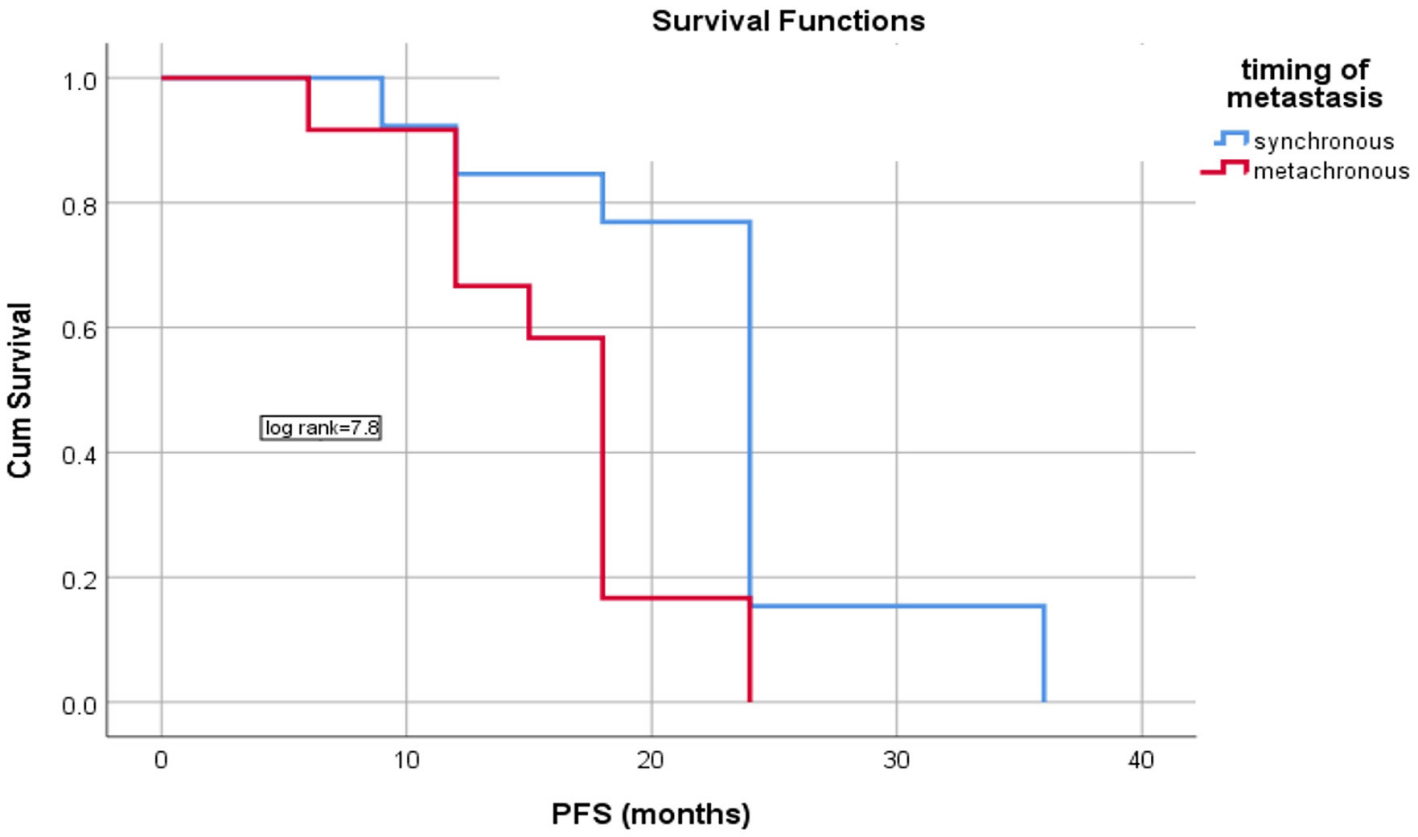
Differences in the PFS according to timing of metastasis.

Unexpectedly, patients with synchronous metastasis who received 6 months of Panitumumab based combinations followed by maintenance Panitumumab/Capecitabine were found to have longer PFS compared to those with metachronous metastasis with mean PFS of 23.3 ± 2.1 and 16.3 ± 1.5 months (95% CI = 19.2–27.4 and 13.3–19.2 months) respectively, *p* = 0.005, Figure 4.

**Figure 5:**
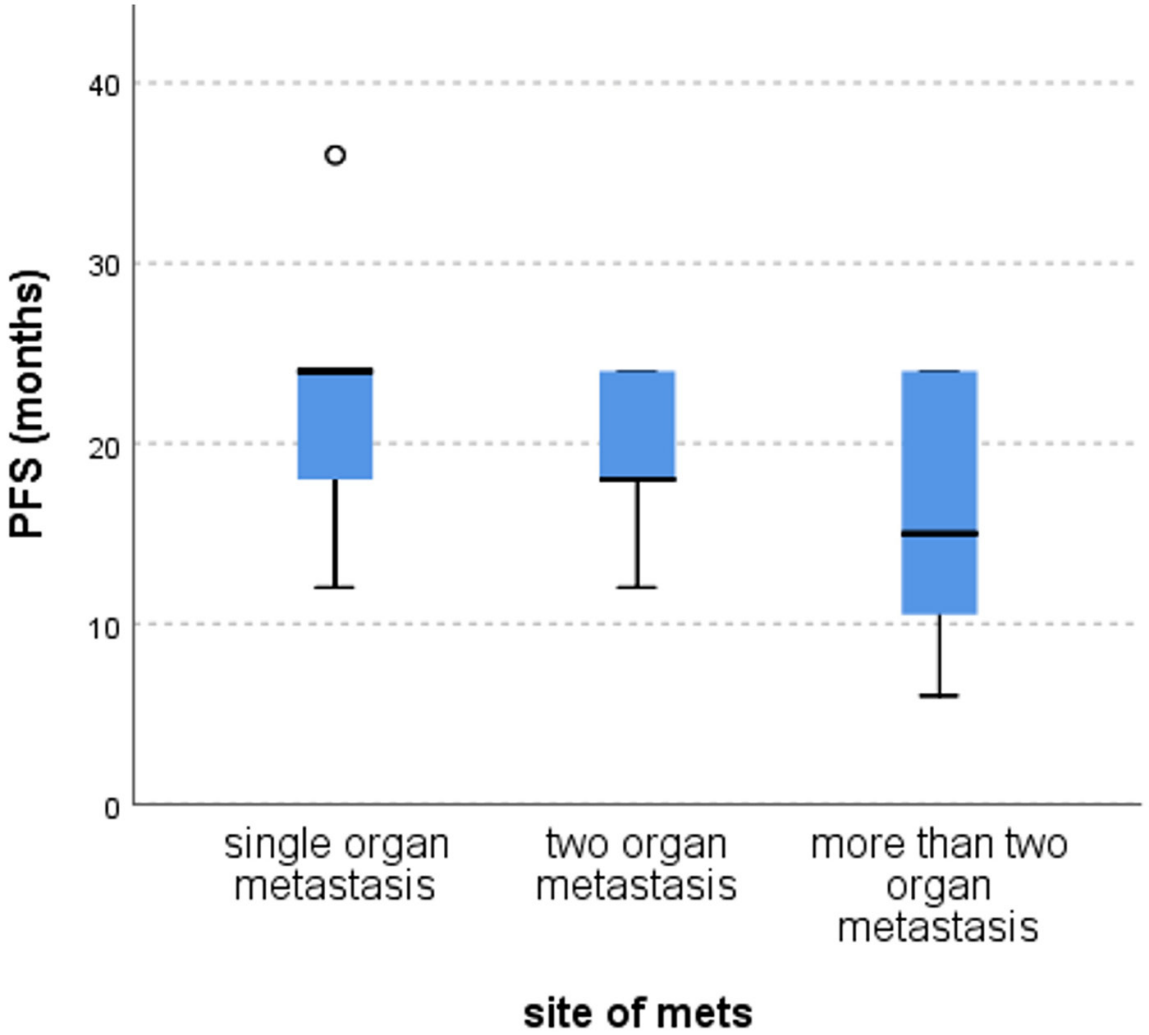
Differences in PFS according to site of metastasis, Kruskal Wallis test, *p* = 0.2.

There was no significant impact of sex (*p* = 0.5), performance status (*p* = 0.4), T staging (*p* = 0.3), N staging (*p* = 0.6), pathology (*p* = 0.9), grade (*p* = 0.053), and primary side (*p* = 0.2). Although there was no significant impact of the number of organs affected by metastasis on PFS, patients with a single organ affected had better PFS compared to those of two or more organs affected (mean ± SD = 22.3 ± 7.5 vs. 19.2 ± 5.02 vs. 16.3 ± 7.7, *p* = 0.2), Figure 5.

**Figure 6:**
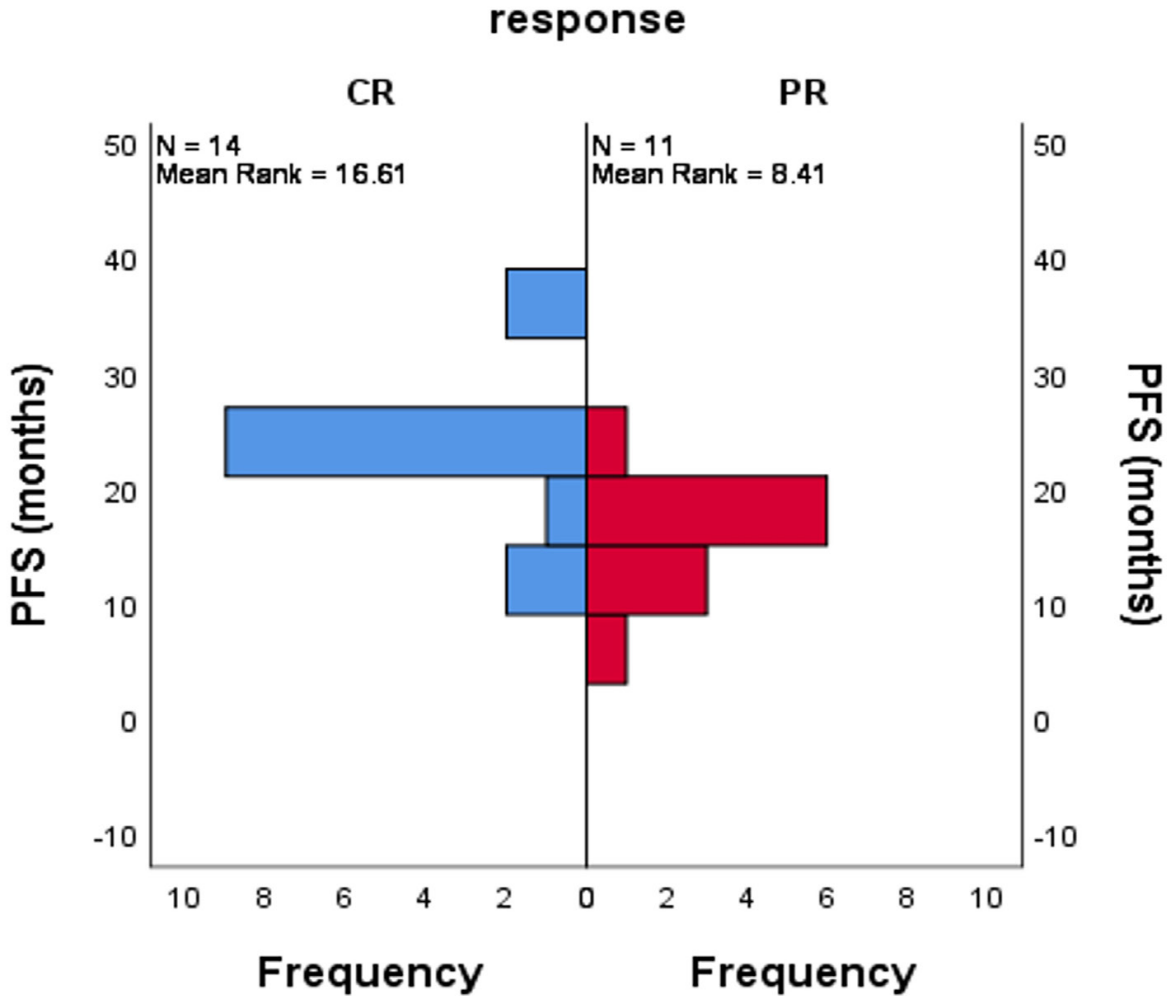
Impact of the response to Panitumumab based combination prolonged PFS after maintenance Panitumumab/Capecitabine, Mann Whitney test, *p* = 0.004.

The distribution of PFS was significantly better in patients achieving CR on Panitumumab-based combination followed by maintenance therapy compared to patients achieving PR on the same regimens. (mean ± SD was 23.4 ± 7.4 vs. 15.6 ± 4.8, *p* = 0.004), Figure 6.

**Figure 7:**
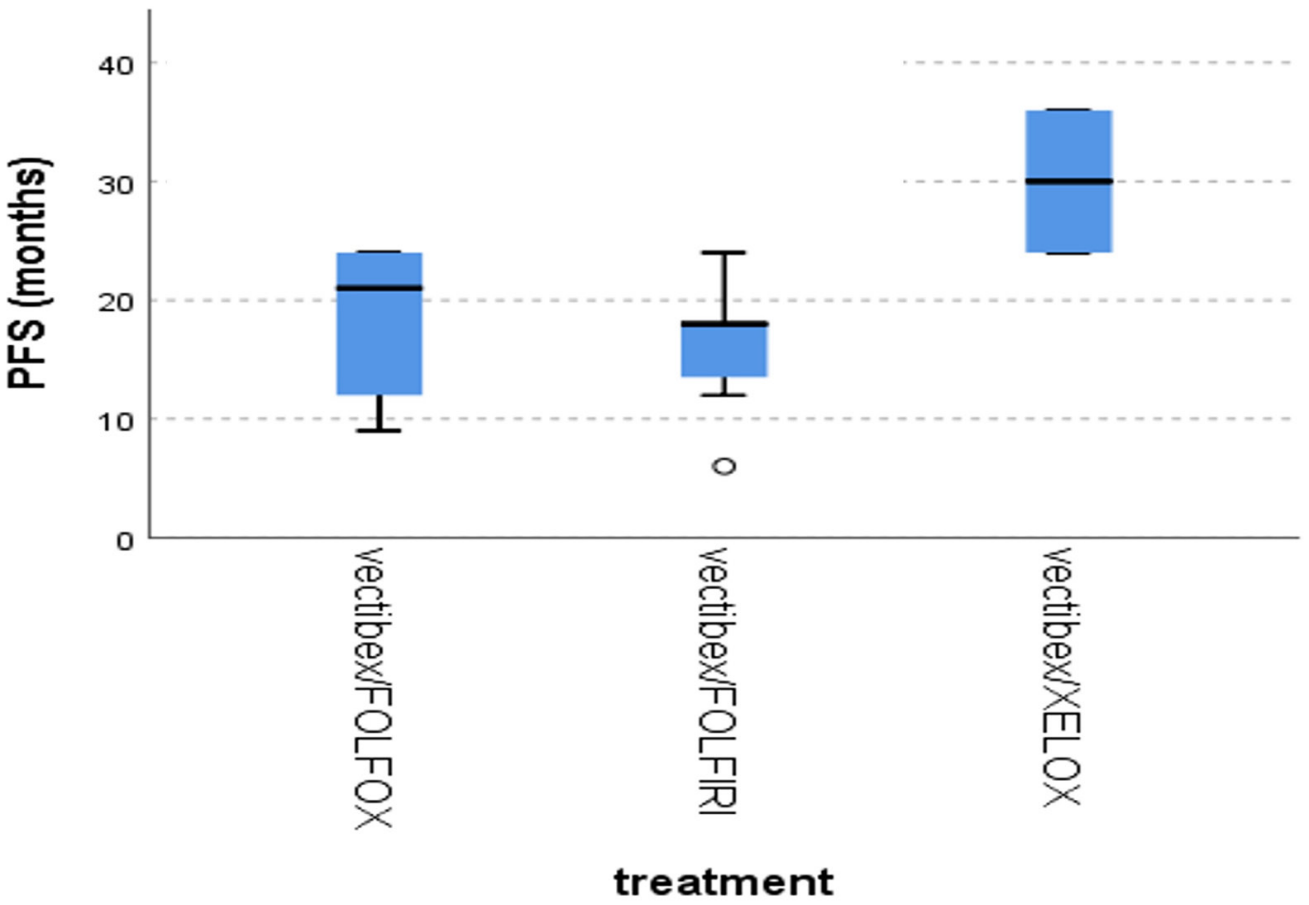
Differences in PFS according to type of treatment, Kruskal Wallis test, *p* = 0.017.

Furthermore, the distribution of PFS was significantly better for those receiving Panitumumab/XELOX compared to Panitumumab/FOLFOX and Panitumumab/FOLFIRI (mean ± SD = 30 ± 6.9 vs. 19.1 ± 5.7 vs. 15.9 ± 5.7 respectively, *p* = 0.017), Figure 7.

### Comparative analysis between synchronous and metachronous metastases

Subgroup analysis of synchronous and metachronous metastasis showed no significant impact of age, sex, PS, pathology, grade, T stages, N stages, and site of metastasis on the PFS difference between both situations; however, all patients with synchronous metastasis had left sided tumor, and 92.3% of the patients with synchronous metastasis received panitumumab/FOLOFOX and panitumumab/Capox regimen which was associated with better responses. Most patients with synchronous metastasis achieved CR (84.6%) compared with 25% in the metachronous group. All these differences could explain why those patients with synchronous metastasis had prolonged PFS, Table 3.

**Table 3:** Subgroup analysis of synchronous and metachronous metastases

Characteristics	Synchronous (13/25)	Metachronous (12/25)	*p*-value
Age	56.7 ± 16.5	52.0 ± 8.02	0.37
Sex			
Male	3 (23.1%)	5 (41.7%)	0.41
Female	10 (76.9%)	7 (58.3%)	
PS			
PS0	6 (46.2%)	9 (75%)	0.32
PS1	5 (38.5%)	3 (16.7%)	
PS2	2 (15.4%)	1 (8.3%)	
Pathology			
Adenocarcinoma	10 (76.9%)	10 (83.3%)	1.0
Mucinous carcinoma	3 (23.1%)	2 (16.7%)	
Grade			
G1	2 (15.4%)	0 (0%)	0.2
G2	6 (46.2%)	8 (66.7%)	
G3	5 (38.5%)	4 (33.3%)	
Site of the primary			
Lt colon	13 (100%)	8 (66.7%)	<0.001
Rt colon	0 (0%)	4 (33.3%)	
T-stage			
T2	0 (0%)	2 (16.7%)	0.16
T3	6 (46.2%)	6 (50%)	
T4	7 (53.8%)	4 (33.3%)	
N-stage			
N0	2 (15.4%)	5 (41.7%)	0.31
N1	7 (53.8%)	4 (33.3%)	
N2	4 (30.8%)	3 (25%)	
Sites of metastasis			
Single organ metastases	9 (69.2%)	4 (33.3%)	0.13
Two organ metastases	1 (7.7%)	4 (33.3%)	
>two organ metastases	3 (23.1%)	4 (33.3%)	
Treatments			
panitumumab/FOLFOX	8 (61.5%)	6 (50%)	0.008
panitumumab/FOLFIRI	1 (7.7%)	6 (50%)	
panitumumab/Capox	4 (30.8%)	0 (0%)	
Response			
CR	11 (84.6%)	3 (25%)	0.005
PR	2 (15.4%)	9 (75%)	

Data expressed as numbers, percentages, and mean ± SD, and analyzed by Pearson Ch^2^ test and Fischer’s exact test.

### Toxicity of protocol

**Figure 8:**
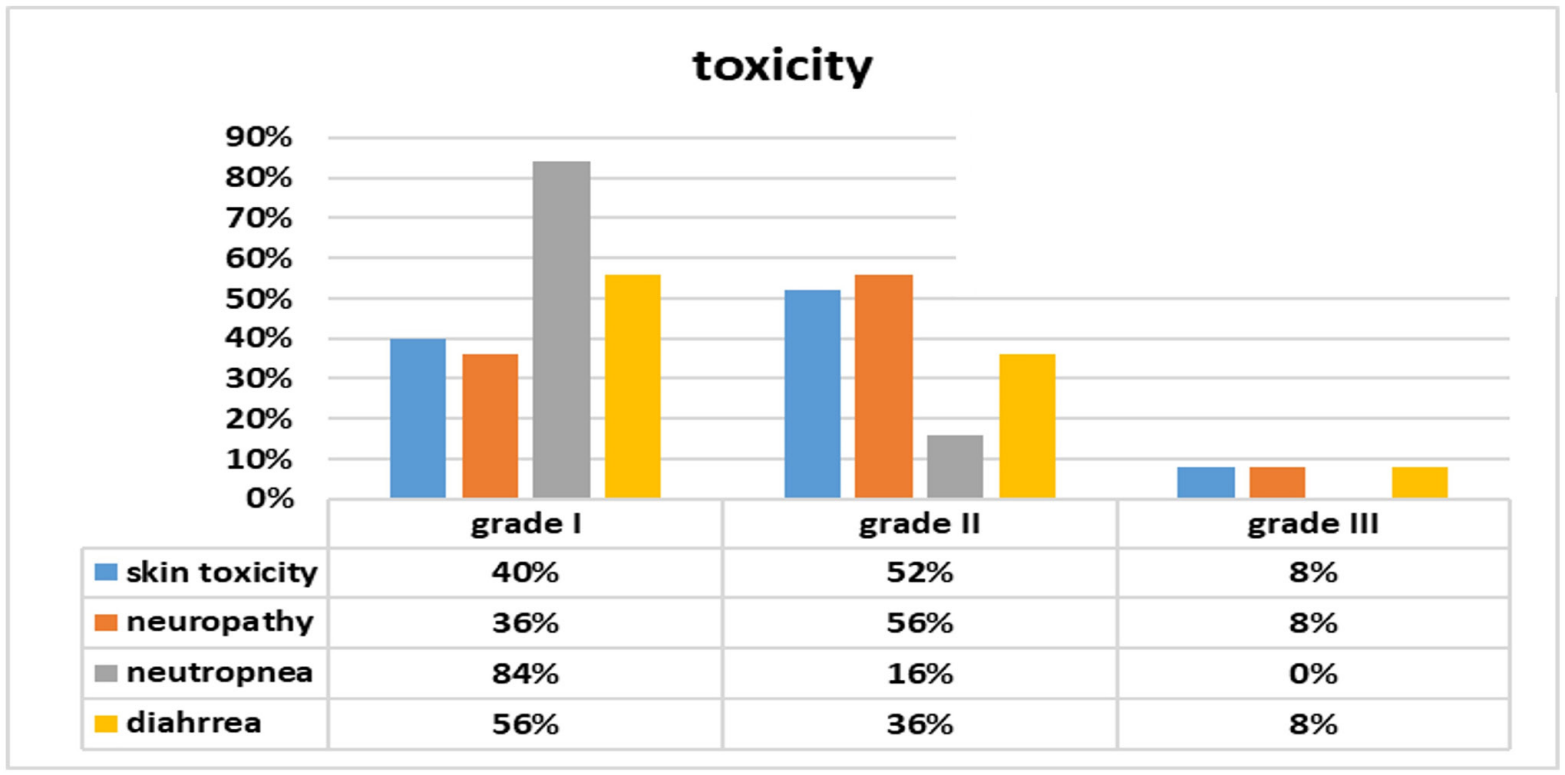
Toxicity profile of maintenance Panitumumab/Capecitabine in 25 patients with advanced CRC.

Maintenance Panitumumab/Capecitabine was tolerable with acceptable grade III toxicities which were reported in only 2 patients (8%) in the form of skin rash and pigmentation and improved considerably on medical treatments. Grade III neuropathy and diarrhea were managed by short-duration treatment interruptions and hospitalization for fluids, antibiotics, neurotropic including pregabalins, and loperamide drug. These toxicities were reverted to grade I-II, and subsequently allowed for the continuation of maintenance therapy even without dose reduction, Figure 8.

## DISCUSSION

Most patients in our study aged more than 50 years, and 20% had mucinous adenocarcinoma in which poor prognosis was expected. All patients except 8 cases were left sided. 56% of the patients had single organ metastasis with 52% having synchronous metastasis. CR rate reached 56% more in patients who received oxaliplatin based therapy. Median PFS was 18 months, and median OS was 45 months. PFS showed significant prolongation reaching 23.3 months in synchronous metastatic patients who received CAPOX and by subgroup analysis these patients with synchronous metastasis had left sided tumor, and 92.3% of patients with synchronous metastasis received Panitumumab/FOLFOX and Panitumumab/CAPOX regimen which were associated with better responses. Most patients with synchronous metastasis achieved CR (84.6%) compared with 25% in the metachronous group. All these differences could explain why those patients with synchronous metastasis had prolonged PFS. There was no significant impact of age, T and N stage and primary sidedness on survival in our study. The principle of combining panitumumab and low dose metronomic capecitabine which refers to continuous administration of low dose cytotoxic drugs without interruption is based on synergistic effect which leads to increased time of the occurrence of resistance to panitumumab.

RuiGeng et al. studied metronomic capecitabine after CAPOX first line treatment in metastatic colorectal cancer compared with observation in responding patients with PFS 5.6 months in metronomic capecitabine arm and median OS reached 23.8 months which was inferior to our results. In this study the number of patients who received metronomic capecitabine as maintenance was 25 patients like our study; however, only 40% had synchronous metastasis and 56% had less than 2 sites of metastasis with diarrhea, neutropenia and hand foot syndrome were the most common toxicities [15].

OPTIMOX trial evaluated maintenance with 5FU/LV, median PFS reached 19.3 months which is better than our results with grade 3–4 toxicity occurring in 48.7% of the patients and this was much higher than grade 3 toxicity in our study [16].

Maintenance with anti-*EGFR* in metastatic colorectal cancer (mCRC) with wild type *Ras* was studied in many trials with promising results*.*

As shown in MACRO-2 trial, results suggested non inferiority of Cetuximab alone versus Cetuximab with mFOLFOX6 in PFS which were inferior to our results, 28% of the patients who received cetuximab only had grade 3–4 neutropenia, and 15% had acne form rash, which increased to 24% when combining cetuximab with FOLFOX [8]. Lu Wang et al. assessed Cetuximab plus Capecitabine as maintenance in responding patients and there was an improvement in one-year PFS reaching 62% with tolerable toxicity profile as only 17% experienced acne form rash and 11% had hand foot syndrome and on that basis our study was built to study if the combination of Panitumumab with low dose Capecitabine is applicable or not regarding toxicity and PFS [4].

The Cairo3 study demonstrated significant PFS improvement with capecitabine and bevacizumab 11.7 months with 23% hand foot syndrome. Our results are much better and more tolerable [7].

In PRODIGE trials which investigated bevacizumab maintenance compared to cetuximab maintenance the median PFS was 9 months and 5.3 months respectively which are inferior to our study [17].

In Valentino trial PFS was better in maintenance Panitumumab with 5 FU/LV than with Panitumumab alone with a slight increase in toxicity and PFS was better in metachronous metastatic patients, the median PFS reached 14.1 months in combination between panitumumab and 5FU/LV with higher but acceptable toxicity occurrence than single panitumumab group [18]. Like Valentino’s and Panama’s [18, 19] studies, our study found that more than half of the patients had synchronous metastasis with significant improvement in PFS in synchronous than metachronous metastasis, but this is not in line with Valentino.

In our research, the toxicity profile was very acceptable, and no patients needed to stop treatment or had a dose modification due to toxicity. MACBETH trial demonstrated 11.2 months PFS in cetuximab single agent maintenance and most patients had synchronous liver single site metastasis while in our trial PFS reached 23 months in synchronous metastasis [20].

We noticed that most patients who got clinical benefits were on CAPOX or FOLFOX plus Panitumumab as initial treatment compared to patients who received FOLFIRI. In addition, patients who received Irinotecan were patients who developed metachronous metastasis or as a second line. PFS in our study was high owing to the high rate of patients entered in complete response and most of them were metastatic in one site and we are not referring to oligometastasis which points to the number of metastases. The tables below summarize comparisons between different trials which discussed the use of different maintenance drugs in metastatic colorectal.

**Table:** 

Study	Current study	RuiGeng et al.	OPTIMOX1	MACRO2
Maintenance given	Panitumumab/ Metronomic capecitabine	Metronomic capecitabine	5FU/LV	armA: cetuximab armB: cetuximab/mFolfox6
Median PFS	18 months	5.66 months	19.3 months	A: 9 months B: 10 months
Median OS	45.8 months	23.82 months		A: 23 months B: 27 months
G3-4 Toxicity	Skin: 8% Diarrhea: 8%	32%	48.70%	A: 70% B: 68%

**Table:** 

Study	VALENTINO	PANAMA	MACBETH
Maintenance given	A: panitumumab/5fu/lv B: panitumumab	A: panitumumab/5fu/lv B: 5fu/lv	A: cetuximab B: bevacizumab
Median PFS	A: 14.1 months B: 10.8 months	A: 8.8 months B: 5.7 months	A: 13.3 months B: 10.8 months
Median OS		A: 28.7 months B: 25.7 months	A: 37.5 months B: 37 months
G3-4 Toxicity	A: 42.4% B: 20.4%	7.20%	Only 3% in either arm.

**Table:** 

Study	LuWeng etal	CAIRO3	PRODIGE
Maintenance given	Cetuximab/ low-dose capecitabine	Bevacizumab/ Capecitabine	A: bevacizumab/5fu based chemotherapy B: cetuximab/5fu based chemotherapy
Median PFS	12.7 months	11.7 months	A: 7.1 months B: 5.6 months
Median OS	27.4 months		A: 15.8 months B: 10.4 months
G3-4 Toxicity	17% acneform rash. 11% hand-foot .4% diarrhea	23% skin toxicity	A: 80% B: 85%

Unfortunately, we could not engage a larger number of patients to participate in this study, which is considered a limitation in our study and this is due to the financial problems that we faced in making drugs available as our patients are not covered by the health insurance system, so we recommend conducting this research on a larger number of patients.

## MATERIALS AND METHODS

It is a prospective one-arm study single-center experience carried out in the Clinical Oncology Department at the Assiut University Hospital from September 2022 to December 2023. The study was approved by Assiut University Ethical Approval Committee, and an informed written consent was taken from all participating patients. Eligible patients had metachronous or synchronous pathologically approved left-sided metastatic colorectal adenocarcinoma wild type *KRAS* and *BRAF*. We enrolled 25 patients. All patients were Eastern Cooperative Oncology Group performance status of (ECOG PS) 0-2, aged 18 years or older.

All patients received 6 cycles CAPOX or FOLFOX or FOLFIRI with Panitumumab (panitumumab, 6 mg/kg (1-hour infusion for the first administration; 30-minute infusion thereafter) and patients who had partial response PR, complete response CR or stationary disease SD received metronomic Capecitabine (one tablet every 8 hours) with Panitumumab 6 mg/kg every 2 weeks till disease progression, intolerable AEs, or occurrence of death.

Our rationale for the chosen Capecitabine schedule is administration of the drug for a long time without drug free break time to try to overcome drug resistance and the decreased dose is due to decreased toxicity and improved patient compliance.

To evaluate the response to metastatic lesions after treatment radiological evaluation was performed every 8 weeks until disease progression or completion of one year of this regimen.

The disease was assessed radiographically according to the Response Evaluation Criteria in Solid Tumors (RECIST), version 1.1. Treatment-related Adverse Events (AE) were graded according to the Common Terminology Criteria for Adverse Events (CTCAE) (version 3). Preemptive treatment of skin rash with doxycycline at the dose of 100 mg/d for 5 consecutive days was given every other cycle starting at cycle1, day1. Grade III neuropathy and diarrhea were managed by short-duration treatment interruptions and hospitalization for fluids, antibiotics, neurotropic including pregabalins, and loperamide drug.

The primary endpoint was progression free survival (PFS) with metronomic Capecitabine and Panitumumab maintenance therapy, while secondary endpoints were toxicity and overall survival (OS).

### Statistics

Both inferential and descriptive statistics were done using IBM-SPSS version 26. All scale variables were non-parametric using the Shapiro-Wilk test. For the relationship of two categorical variables, the Chi^2^ test was used. However, when variables had ≥25% table cells with counts ≤5, likelihood ratio was used, for the relation between categorical and continuous variables, Mann Whitney *U*-test, Kruskal Wallis test, and log-rank tests were used.

Progression-free survival (PFS) was the time elapsed between the start of the first- line treatment for synchronous and metachronous metastatic tumors and the development of an event including death, progression, or missing.

Overall survival (OS) was defined as time passed from the start of the first-line treatment either as an adjuvant or for synchronous metastatic tumors to death or missing.

Survival curves were analyzed using the Kaplan-Meier test, and all data were considered significant at *p* ≤ 5%.

## CONCLUSIONS

Using Panitumumab with metronomic Capecitabine is considered an accepted maintenance regimen in wild Ras metastatic colorectal cancer regardless of the site of primary and the most preferred regimen to be given to patients who received Oxaliplatin based chemotherapy/Panitumumab regimen with synchronous metastasis.
